# Implementation of a Primary Prevention Program for Posttraumatic Stress Disorder in a Cohort of Professional Soldiers (PREPAR): Protocol for a Randomized Controlled Trial

**DOI:** 10.2196/47175

**Published:** 2024-01-26

**Authors:** Emmanuelle Le Barbenchon, Marion Trousselard, Sonia Pellissier, Mathilde Moisseron-Baudé, Philippine Chachignon, Pierre Bouny, Emma Touré Cuq, Sandrine Jacob, Cécile Vigier, Maud Hidalgo, Damien Claverie, Anais M Duffaud

**Affiliations:** 1 Laboratoire de Psychologie Sociale Aix Marseille Université Aix-en-Provence France; 2 French Armed Forces Biomedical Research Institute Brétigny-sur-Orge France; 3 Univ Savoie Mont Blanc, Univ Grenoble Alpes, LIP/PC2S Grenoble France; 4 School of Practicing Psychologists Paris France; 5 VCR Team, School of Practicing Psychologists Catholic Institute of Paris - Religion, Culture and Society Reception Team Paris France; 6 Center for Research on Work and Development Conservatoire National des Arts et Métiers, EA4132 Paris France; 7 URGOTECH Paris France; 8 Centre d'Epidémiologie et de Santé Publique des Armées Marseille France

**Keywords:** posttraumatic stress disorder, military, primary prevention, biopsychosocial, resilience, coping, stigma, biophysiology, PTSD, implementation, soldier, veterans, prevention program

## Abstract

**Background:**

Posttraumatic stress disorder (PTSD) is a psychiatric disorder that can manifest after a traumatic event where the individual perceives a threat to his or her life or that of others. Its estimated prevalence in the European population is 0.7% to 1.9%. According to the “dose-response” model, individuals who are most exposed to traumatic events are most at risk of developing PTSD. Hence, it is unsurprising that studies have observed a higher prevalence among the military population, ranging from 10% to 18%, or even up to 45%. This project’s overall goal is to evaluate the primary prevention actions that can strengthen the resilience of at-risk professionals, notably military personnel, in the short term, with the medium- to long-term aim of preventing the occurrence of PTSD and improving the patient’s prognosis.

**Objective:**

This study’s objectives are (1) to design a primary prevention program for PTSD, tailored to the studied military population and compatible with operational constraints; and (2) to implement and validate the Primary Prevention of Posttraumatic Stress Disorder in Military Professionals (PREPARE) program in the short term with operational personnel belonging to the French Mountain Infantry Brigade.

**Methods:**

This is a single-center, prospective, randomized, parallel-group controlled cohort study. The cohort is divided into 2 groups: the nonintervention group receives no training, and the intervention group follows a dedicated prevention program (structured into 8 workshops and 2 debriefing and practice reinforcement workshops). Each participant is evaluated 4 times (at inclusion, +4 months, +6 months, and +12 months). During each visit, participants complete several psychosocial questionnaires (which take 15-80 minutes to complete). Samples (a 30-mL blood sample and three 5-mL saliva samples) are collected on 3 occasions: at inclusion, +4 months, and +12 months. Emotional reactivity (electrocardiogram and electrodermal activity) is measured before, during, and after the classic and the emotional Stroop task.

**Results:**

The project is currently ongoing, and results are expected to be published by the end of 2024.

**Conclusions:**

The study adopts an integrative approach to the processes that play a role in the risk of developing PTSD. Our biopsychosocial perspective makes it possible to target levers related to factors specific to the individual and socio-professional factors. The following dimensions are addressed: (1) biophysiology (by studying markers of the neurobiological stress response, wear and tear, and vulnerability phenomena and reinforcing the flexibility of the autonomic nervous system), (2) psychology (by facilitating and measuring the development of flexible coping strategies to deal with stress and evaluating the moderating role of the individual’s sense of duty in the development of PTSD), and (3) social (by facilitating community strategies aimed at reducing stigmatization and supporting the use of care by professionals in difficulty, in the institutional context).

**Trial Registration:**

ClinicalTrials.gov NCT05094531; https://clinicaltrials.gov/study/NCT05094531

**International Registered Report Identifier (IRRID):**

DERR1-10.2196/47175

## Introduction

### Background and Rationale

#### Overview

Posttraumatic stress disorder (PTSD) is a psychiatric disorder that manifests following the experience of a traumatic event (TE) where the individual has perceived a threat to his or her life or that of others [1]. In France, it is considered to be the third most widespread psychiatric disorder, after major depression and specific phobias. Its prevalence in the European population is estimated to be between 0.7% and 1.9% [2,3]. According to the “dose-response” model, individuals who are most exposed to TEs are most at risk of developing PTSD [3-5]. At-risk occupations, such as the military, law enforcement, and first responders, carry an inherent risk of experiencing trauma. Hence, it is unsurprising to observe a high prevalence of PTSD in military populations, ranging from 10% to 18% or reaching up to 45% [5-9], depending on the study. Although cumulative exposure seems to be an important determinant in the general population [10], the literature does not establish a clear link among at-risk professionals. Nevertheless, it is reasonable to think that cumulative exposure is detrimental, and may even lead to fragilization, which would be consistent with the intensity of PTSD symptoms [11,12]. Repeated exposure triggers a reaction that builds on the consequences of previous exposures, increasing the complexity of the link between cumulative exposure and PTSD development, via multiple pathways.

#### PTSD Prevention

New medical research has influenced public policy, and the promotion of good health has become a key priority where policies seek to encourage certain behaviors that protect, improve, or restore the health of individuals, groups, or the entire population, while a prevention framework is used when the goal is to limit or prevent the development of a specific disease.

In the 1980s, evidence-based medicine emerged. The approach is founded on the principle of structuring prevention and public health decisions based on scientific evidence (eg, epidemiological studies and systematic clinical studies). A given health problem can be addressed by preventive interventions at primary, secondary, or tertiary levels. The objective of primary prevention is to intervene before the problem occurs; actions target the determinants of the problem by reducing risk factors or by promoting or reinforcing protective factors. In the context of PTSD, primary prevention can intervene at the level of preventing the trauma or at the level of PTSD prevention after the TEs. For at-risk professions in particular, PTSD prevention is the primary target, as the risk of exposure to trauma is integral to the profession. Secondary prevention seeks to define interventions once the problem is identified; it consists of early detection and referral for treatment (ie, after exposure to a TE). Finally, tertiary prevention seeks to manage the problem and prevent relapse. If necessary, the goal is to treat or limit any aggravation of the problem, notably any psychosocial consequences or comorbidities associated with PTSD. At all 3 levels, prevention must respect individual freedom [13].

#### Primary Prevention of PTSD

Effective primary prevention relies upon a sound theoretical understanding of the processes and determinants of the problem, knowledge of actions that have been scientifically validated in other populations, and clinical expertise in the field, which reflects the characteristics (eg, sociocultural) of the target population. This “selective prevention” approach seeks to target exposed participants.

In the domain of PTSD, primary interventions are rare, as such actions aim to prevent the impact of TEs before they occur. Nevertheless, this literature [14] gives us an insight into the potential effects of cumulative exposure to TEs during the career of military professionals. In this context, primary prevention interventions target several determinants that contribute to developing resilience following the TE. The approach underlines the need to characterize the factors that support individual and collective resilience processes, tailored to the characteristics of the population of at-risk professionals. This initial step is a prerequisite to the definition of relevant targets and the development of an appropriate prevention intervention for the population.

The proposed program (which we call Primary Prevention of Posttraumatic Stress Disorder in Military Professionals [PREPAR]) is part of a comprehensive prevention approach that synergistically links physiological, psychological, and social determinants. The approach stems from the etiological model proposed by Jones and Barlow [15]. This model is a comprehensive framework used in clinical psychology to understand the development and maintenance of psychological disorders, particularly PTSD. It integrates various factors that contribute to the onset and perpetuation of these disorders. Several etiological factors, including biological, cognitive, and behavioral components, are implemented in this model. The model also takes into account predisposing factors and moderating variables. It emphasizes a holistic approach to understanding mental disorders, taking into account the complexity of the human experience.

#### The Physiological Dimension

PTSD is described as a failure of emotional extinction which develops following exposure to intense fear. Exposure to a stressful event leads to a neurobiological stress response, resulting in the activation of the sympatho-adrenergic neurovegetative system and the corticotropic neuroendocrine axis. Although this dual physiological response is effective in the short term, it comes with a biological cost; regulatory mechanisms aim to compensate for this loss by supporting poststress recovery. A repeated inability to recover and extinguish the stress response in the long term (repeated exposures over a short time period) creates a so-called “allostatic load” [16], which progressively limits the flexibility of the central and peripheral nervous system. The peripheral nervous system is of particular interest, due to its role as a mediator of the allostatic load. The parasympathetic branch plays a role in the emotional extinction and correction of the load, making it a key vulnerability factor for health when it is insufficiently effective [17]. Certain professions (frontline responders and military personnel) are at risk of developing a significant allostatic load, due to their repeated exposure to TEs (inherent in the nature of their work). On top of this, personnel must adapt, on a daily basis, to various environmental demands: physiological (sleep debt, altered sleeping patterns, etc), physical (hypoxia, etc), and cognitive and emotional (traumatic exposure, etc), among others. All of these factors test the flexibility of the individual’s physiological systems, particularly the autonomic (ie, parasympathetic) nervous system, which, consequently, appears to be a key target for prevention measures.

#### The Psychological Dimension

Military personnel participate in multiple missions in conflict zones, and this is likely to alter the flexibility of their executive and emotional regulation functions. Although training programs aim to develop automated responses to well-known mission scenarios, they do not focus on developing the change in viewpoint that contributes to the resilience process. Moreover, preclinical data show that both acute and repeated stress are likely to reduce cognitive flexibility [18,19]. A slight reduction in cognitive flexibility likely reduces the flexibility of emotional regulation strategies following a TE, thus increasing the impact of exposure. This hypothesis draws upon the notion of “coping flexibility.” The latter concept represents the individual’s ability to evaluate the effectiveness of his or her strategies for coping with stress in a specific situation and then adopting alternative strategies, if necessary [20]. The evaluation of training in the diversification of emotional regulation strategies in military populations has found a reduced risk of PTSD after exposure to TEs. Furthermore, a relationship has been identified between cognitive flexibility and self-compassion [21]. The data also suggest that increasing self-compassion contributes to increasing cognitive flexibility [22].

Finally, a clear sense of duty has been found to counterbalance perceived constraints associated with the mission [23,24]. The literature reports that the meaning attributed to the work environment can, to a significant degree, compensate for the perception and impact of occupational stressors [25].

#### The Social Dimension

The social dimension targets normative pressure and stigmatization. These determinants are specific to the institutional context of military personnel and may represent risk factors. Thus, normative group pressure to follow codes that encourage the nonexpression of emotions [26] can be a risk factor, as military personnel can be reluctant to speak about their psychological and somatic symptoms, which delays treatment. A fear of stigmatization by the institution and peers is another potential barrier to care. Overall, these social factors are obstacles to both individual and collective positive health behaviors.

### Research Hypotheses

#### Overview

The goal of this project is to evaluate a multidimensional biopsychosocial primary prevention intervention for PTSD aimed at strengthening the resilience of at-risk professionals, namely military personnel, in order to prevent the occurrence of PTSD or reduce its severity.

The objectives of the project are as follows: (1) to design a primary prevention program for PTSD specific to the studied military population and compatible with operational constraints; (2) to implement or validate the program with operational personnel belonging to the French Mountain Infantry Brigade (*Brigade d’Infanterie de Montagne*); and (3) to understand PTSD and its prevention from 3 perspectives: biophysiological (by studying key markers of the neurobiological stress response, strain and vulnerability and increasing the flexibility of the autonomic nervous system); psychological (by facilitating and measuring the development of flexible strategies to cope with stress and evaluating the moderating role of the meaning of the mission in the development of PTSD); and social (by facilitating community strategies aimed at reducing stigmatization and helping professionals in difficulty to access care in the institutional context).

Our biopsychosocial approach adopts an integrative understanding of the processes at play in the risk of developing PTSD. This perspective makes it possible to target levers related to factors specific to the individual (at physiological and psychological levels), and contextual and social factors (related to the working environment).

#### The Physiological Dimension

The change in parasympathetic vagal flexibility is an early and silent sign of physiological deterioration. It can be identified by noninvasive measurements (resting or tonic heart rate variability, and activation or phasic heart rate variability) and modulated by exercises that target vagal activity (cardiac coherence techniques), which can be easily integrated into a busy professional agenda. A preliminary feasibility study among a group of firefighters (SDIS 73) demonstrated very good acceptance of this technique by the selected professionals and good adoption in daily life [24].

#### The Psychological Dimension

Two factors are targeted in the psychological dimension: coping flexibility and the sense of duty. These 2 determinants have rarely been targeted in studies of the prevention of occupational PTSD. The focus on coping flexibility and self-compassion seeks to improve emotional regulation, and the focus on the sense of duty seeks to target the silent determinants of occupational PTSD, with the overall aim of identifying an effective primary prevention intervention.

#### The Social Dimension

The targeted determinants are the beliefs and socio-normative processes that play a role in PTSD in a military population (self-stigmatization, stigmatization by others, normative pressure, and group cohesion). The intervention aims to establish a dedicated space to normalize the discourse, with the overall aim of discussing PTSD and making it visible, along with the signs of emerging psychological and somatic injuries. Specific group facilitation techniques are used, notably social modeling, which aims to reinforce feelings of self-efficacy that support the public expression of signs of injury and to support the search for appropriate care, when necessary [27].

### Objectives of the Research

The main objective is to determine the effectiveness of a biopsychosocial program targeting the resilience (developing stable resources to adapt to occupational demands, in order to cope with TEs) of military personnel in the context of PTSD prevention.

The secondary objective is to better understand interindividual variability regarding the program’s impact. We will study levels of vulnerability at inclusion and their impact on the program’s effectiveness. We will also track postprogram changes in resilience (at 6 and 12 months). In addition, we will evaluate (1) the impact of the program on activation and psychobiological deterioration biomarkers and (2) the psychosocial determinants at 4 months (the end of the program) and 12 months (to evaluate persistence). In addition, we will measure adherence to the intervention at group and individual levels, through an evaluation at the end of the program. Finally, we will examine user satisfaction with the URGOfeel sensor and its application during workshops.

## Methods

### Ethical Considerations

Prior to their participation in the study, all participants will receive 2 separate notifications: one detailing the primary study and the other focusing on the genetic part. It is essential to emphasize that participation in the genetic segment is entirely optional, allowing individuals to engage solely in the main study if they prefer. Both of these notifications provide comprehensive information about the study’s objectives, limitations, and legal requirements, especially in terms of privacy and confidentiality. Additionally, they clearly outline the potential benefits and risks associated with participation.

All collected data will undergo rigorous anonymization and processing in strict accordance with the MR001 reference methodology, in compliance with the regulations of the French Data Protection Authority (CNIL).

Our study maintains a steadfast commitment to ethical standards, in alignment with the principles set forth in the 1964 Helsinki declaration and its subsequent amendments. Furthermore, the ethics committee of Ile de France 8 formally approved the research protocol on January 12, 2021 (reference 20.12.10.58611). It is important to note that participants will not receive any form of compensation for their participation in this research.

### Recruitment

The study will be conducted with members of the 27th Mountain Infantry Brigade, located in the Auvergne-Rhône-Alpes region of France. Participants will be recruited from companies proposed by the Brigade’s line manager, depending on the ability to operationalize and implement measurement sessions, and the intervention.

### Randomization

Individual randomization is not feasible for two reasons: (1) the logistics involved in deploying the training program and the availability of participants; and (2) the need to avoid a contagion effect between members of the Brigade who benefit from the program and those who do not.

### Eligibility Criteria

After obtaining informed, written consent, the following inclusion criteria will be verified: affiliated to a social security scheme; a male adult; a member of a combat unit with external operations (OPEX) capability; a soldier with a current contract with the Brigade, lasting a minimum of 12 months; a soldier who is able to attend all measurement sessions and workshops, according to the schedule defined upstream; a soldier who is not a member of the *Groupements Commando Montagne* (to ensure homogeneity); and, finally, nonparticipation in one of the studies included in phase 1 of the PREPAR project. The latter social psychology study aimed to understand the day-to-day experience of frontline professionals and was approved by the ethics committee of the University of Aix-Marseille (2019-12-12-001). Noninclusion criteria are as follows: being female; receiving treatment for a chronic disorder (daily medication for at least 1 month); participation in an external operation planned within 12 months; and being an adult ward of court.

### Participant Timeline

**Figure 1:**
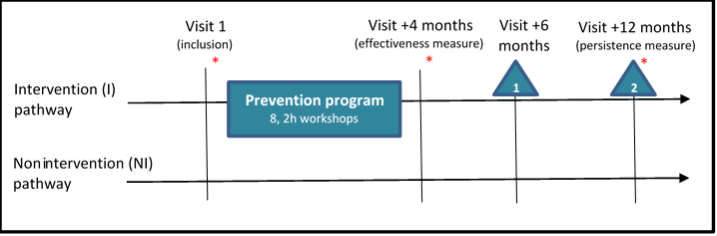
Study timeline. *: 2 to 3 hours to take measurement; △: “debriefing and anchoring of the practice” workshop.

Figure 1 presents the timeline for participants, and Table 1 presents a synopsis of how the study will unfold.

**Table 1:** Summary of the stages of the study.

	Visit 1 inclusion	+4 months	+6 months	+12 months
Information – consent	✓			
Sociodemographic data	✓			
Self-administered questionnaires	✓	✓	✓	✓
Blood and saliva collection (physio-biology and genetic)	✓	✓		✓
Emotional reactivity	✓	✓		✓
“Debriefing and anchoring of the practice” workshop			✓	✓

The first step is a briefing session with 2 of the Brigade’s companies. Each individual will receive 2 information letters (1 regarding participation in this study, and 1 regarding participation in genetic studies), in order to allow time to reflect before the inclusion visit.

### Visit 1: Inclusion

Inclusion will take place over a 3-week period. This time is needed to be able to disseminate information to the groups concerned and collect data. Once informed consent has been obtained, and eligibility criteria have been verified by one of the investigators, the following samples will be collected: (1) a 30-mL blood sample; (2) three 5-mL saliva samples (the participant will be asked to collect a sample on waking, getting up, and 30 minutes later [28]); (3) noninvasive measurements of cardiac variability and electrodermal conductance (using patches applied to the skin) during Stroop tasks, estimated to take 20 minutes; and (4) a psycho-cognitive evaluation (standardized and validated questionnaires). The latter will make it possible to evaluate the psychological and somatic symptoms (Patient Health Questionnaire [PHQ-15] [29]; psychosocial factors; Perceived Stress Scale [PSS] [30]; coping flexibility [20]; inner correspondence and peaceful harmony [ICPH] [31]; stigma and barriers to care [32]; Multidimensional Scale of Perceived Social Support [MSPSS] [33]; the Siebold vertical and horizontal cohesion questionnaire [34]; and the presence of any psychopathologies such as anxiety and depression: Hospital Anxiety and Depression Scale [HADS] [35,36], Posttraumatic Checklist-5 [PCL-5] [37], unsure, and Burnout Measure Short Version [BMS] [38,39]). The time required to fill out the questionnaires is estimated to be 45-60 minutes (184 items in total).

### The Prevention Program: Workshops

Members of the intervention group will participate in 8 workshops. Each lasts 2 hours and is divided into three parts: (1) welcome participants with time allocated for inclusion (time approximately 20 minutes); (2) a presentation of the pedagogical objectives of the session, and its practical application; and (3) group wind-up (approximately 20 minutes). Please see Multimedia Appendix 1 for a description of the workshop program. At the end of the study, members of the nonintervention group, who wish to do so, will be able to benefit from the same prevention program during a later session, which will be scheduled as a function of operational constraints.

### Visit +4 Months: Follow-up

A second round of data collection will take place at the end of the prevention program (approximately 4 months after inclusion). Each participant will be asked to allocate half a day to the collection of biopsychological measurements.

### Visit +6 Months: Follow-up

Two months after the end of the program, during the first debriefing workshop, participants will be asked to complete a set of self-administered questionnaires (the same set as the one completed during the inclusion visit a part for the sociodemographic information).

### Visit +12 Months: End of Study

Eight months after the end of the prevention program, each participant will take part in a final measurement session (lasting 2-3 hours). They will also be asked to allocate another half a day to the collection of biopsychological measurements.

Measurements recorded at +4 and +12 months are identical to those performed at inclusion, except for sociodemographic data, and the sample needed to assess vulnerability (genetic polymorphism).

Thus, all participants are required to attend 4 measurement sessions: visit 1, inclusion; +4 months; +6 months; and +12 months. During each of these visits, they will be asked to complete a self-administered (using a tablet) questionnaire to collect psychosocial data. In addition, at inclusion, +4 months, and +12 months, (1) biological samples will be collected for each participant (blood and saliva) and (2) activation of the autonomic nervous system (heart rate variability and electrodermal activity) will be measured at rest, during emotional activation (the classic, then the emotional Stroop task), and at recovery.

### Outcomes

#### Primary End Point

There is no consensus in the literature regarding the definition of resilience, and there are references to several dimensions. Consequently, measuring the effect of the intervention on resilience requires the use of several indicators. A simple approach would be to measure PTSD symptomatology reported by participants, with the expectation that the intervention will result in a decrease in symptoms. Although this approach would be relevant for long-term measures [40], using this criterion in a short-term study, such as ours, would be restrictive and fail to support the processes that are assumed to be activated during the intervention [41]. The latter consists, in part, of removing barriers to seeking care when necessary, in order to support the building of resilience as a process, and not simply as a state. Our short-term approach is also justified by the difficulty of conducting studies in a military environment over the long term (personnel are transferred every 3 years) and attrition among professional soldiers.

The proposed composite criterion groups indicators of resilience that are described as protective factors for PTSD and are recognized to be sensitive to interventions. The first is the participant’s emotional state. It is known to be highly impacted in PTSD, where there is an increase in negative emotions. At the same time, it is a marker of resilience in cases where emotions become positive following trauma. We will therefore use the Positive and Negative Affect Scale (PANAS) score [42], which measures the intensity of positive and negative emotional states. Our second indicator measures self-compassion, which refers to a general tendency to be kind to oneself, despite knowing one’s failures and successes. Self-compassion is very directly linked to the process of self-acceptance and is a predictive criterion for both the development of PTSD (when it is deficient), and increased resilience (when stimulated by a psychotherapeutic intervention). Self-compassion also contributes to cognitive flexibility. Finally, the third indicator is the hardiness score. This measure is classically used in the literature to study, from a psychological point of view, an individual’s ability to remain in good health under stressful conditions. It should be noted that this measure should not, by itself, be considered a sufficient criterion for resilience in our protocol, given that any modification is observed over the long term, based on a quasi-dispositional approach.

Consequently, the hardiness score will be combined with mood and self-compassion criteria. The composite criterion will be evaluated at 4 months (Figure 1). A change in resilience will be defined as favorable if at least two of the following three criteria are met: (1) a 20% improvement in the PANAS score; (2) given the military context, and the nonspecific nature of our intervention, a 20% improvement in scores on the Self-Compassion Scale; or (3) a 5% change in hardiness, measured using the Dispositional Resilience Scale (DRS-15) [43]. If these criteria are not met, any change will be considered to be unfavorable. The choice of thresholds for variables making up the composite criterion is based on data from research conducted by the project’s teams.

#### Emotional State

The data based on the STEP study, carried out in the framework of Delphine Traber’s thesis [24], show a 33.68% long-term increase in the PANAS score among members of the *Bataillon de Chasseurs Alpins* who had undergone training (pretraining score=12.32, posttraining score=15.21, and 6-month posttraining score=16.47). Thus, in the PREPAR study, we consider a 20% improvement as significant (for questionnaire details, see Multimedia Appendix 2 [42-44]).

#### Self-Compassion

According to Kotsou and Leys [44], the mean score is 2.88 in the French population. A score between 3.5 and 5.0 indicates a high level. Thus, in the context of this study, an increase of 20% (0.62) will be considered relevant (for questionnaire details see Multimedia Appendix 2).

#### Hardiness, the Ability to Stay Healthy Under Stressful Conditions

In the literature, global hardiness scores (measured by the DRS-15 scale) in military populations are around 29 (scores can range from 0 to 45). To the best of our knowledge, there are no studies that have evaluated the effect of a prevention program on this variable. Thus, as this is a dispositional measure, we consider a 5% increase as relevant (for questionnaire details see Multimedia Appendix 2).

### Secondary End Points

The evaluation criteria used to meet the secondary objectives are provided in the following sections.

#### Objective 2.1: Vulnerability

We will assess the following regarding vulnerability:

1. Innate: based on a study of the polymorphism of genes involved in stress regulation mechanisms (see Multimedia Appendix 3). These analyses will not be used in a diagnosis.
2. Acquired: based on miRNAs that target regulatory phenomena established in earlier work (see Multimedia Appendix 4.
3. General psychological and somatic symptomatology (the PHQ-15, HADS, and PCL-5).

#### Objective 2.2: Change in Resilience

We will assess follow-up of the change in scores on composite end point questionnaires: PANAS, Self-Compassion Scale, and DRS-15 at 6 and 12 months after inclusion.

#### Objective 2.3: Change in Psychobiological Biomarkers of Activation and Deterioration

We will assess the following regarding change in physiobiological biomarkers of activation and deterioration:

1. Indirect markers of oxidative stress: lipoperoxidation markers thiobarbituric acid reactive substances and 8-iso-prostaglandin F2alpha [45,46].
2. Circulating markers of central nervous system activity: GABA, brain-derived neurotrophic factor, kynurenic acid, and dopamine [47-49].
3. Inflammation: proinflammatory cytokines (including C-reactive protein, TNF-alpha, IL23, and IL12), anti-inflammatories (IL10 and IL6), and chemokines [50,51].
4. Hypothalamic-pituitary-adrenal axis: cortisol (analysis of saliva on waking, getting up, and 30 minutes later), catecholamines, and neuropeptide Y [52].
5. Markers of physiological activation (autonomic nervous system): cardiac variability index (temporal, frequency, and nonlinear analysis of the electrocardiogram signal to measure indices of parasympathetic flexibility), and electrodermal conductance (level of tonic activity, and amplitude of phasic activity to assess activation of the sympathetic system and its persistence) [53,54].
6. Psychological: follow-up of perceived stress (Cohen questionnaire) and psychological symptomatology scores (the PCL-5, the HADS, and the BMS).

#### Objective 2.4: Change in Psychosocial Factors

We will assess the following regarding change in psychosocial factors:

1. Flexibility of coping strategies, Flex Cop [55].
2. Sense of duty, inner correspondence, and peaceful harmony (ICPH) questionnaire [31].
3. Emotional reactivity (scores on the emotional Stroop task are compared with the classic Stroop task before and after the intervention at 4 and 12 months) [56,57]. The aim of this task is to evaluate the flexibility of sympathetic and parasympathetic systems inherent in the emotional response to trauma. The classical Stroop task (baseline) will be followed by the emotional Stroop task (reactivity). No learning effect has been documented with using this experimental modality.
4. Self and public stigma scores of PTSD in the military.
5. Perceived social support (the multidimensional scale of perceived social support) [33], and institutional support (the Sieblod vertical and horizontal cohesion questionnaire).

#### Objective 2.5: Describe Changes in Adherence to the Intervention

Adherence will be assessed using quantitative criteria at 4, 6, and 12 months (the Treatment Motivation Questionnaire). This analysis will be based on (1) an analysis of the processes put in place to operationalize the intervention; (2) a focus group consisting of 8 participants, and 10 individual interviews carried out at 4 and 12 months after inclusion; and (3) a qualitative analysis of discussions that took place during workshops to anchor the practice held at 6 months and 1 year. The latter will also make it possible to identify both obstacles and drivers of adherence.

#### Objective 2.6: Describe User Satisfaction With the URGOfeel System

We will assess user satisfaction with the URGOfeel (Urgothech) application using visual analog scales, and short questionnaires that measure (1) the user experience and (2) its benefits.

### Ancillary Study

We plan to run a qualitative analysis of the transferability of the intervention. The transferability assessment will be carried out at the very end of the research protocol (1 year after inclusion) and will use the transferability and support to the adaptation of health promotion interventions ASTAIRE tool. The aim is to facilitate the transfer of the intervention to other populations, both military and civilian. The intervention could then be tailored to other populations, based on this evaluation. The ancillary study will focus on qualitative details, notably the conditions, obstacles, and drivers facilitating the transferability of the intervention to other populations or other contexts. The criteria required by ASTAIRE will be supplemented by a qualitative evaluation based on interviews (n=20), and a focus group (n=8) at the end of the intervention. To limit bias, we will ensure that participants in the various segments of this ancillary study (ASTAIRE, focus groups, and interviews) are distinct from one another. A summary of the analysis of these criteria (qualitative, Astaire, and quantitative, in the long term) could be used as a guide for future deployments of the intervention.

### Statistical Methods

Continuous variables will be presented as mean and SD, if the distribution is normal (the Shapiro-Wilk test will be used, if necessary). In case of a nonnormal distribution, data will be presented as median, quartiles, and extreme values. Scale variables will be treated as ordinal data and analyzed as above. Depending on the analysis, data may be classed as categorical variables. Categorical variables will be expressed as absolute values and percentages. To control for attrition, calculations will assume 20% of data are missing.

The primary analysis will examine the effectiveness of the prevention program. This will be evaluated by comparing the percentage of responses between intervention and nonintervention groups, using a *χ*^2^ test. Secondary analyses will examine the abovementioned markers. Values for the intervention and the nonintervention group will be compared using a repeated measures ANOVA if the conditions for applying an ANOVA are met. Vulnerability will be initially identified using a cluster analysis of the measured biological variables (a *k*-means or Gaussian model mixture, depending on the distribution). The analysis will seek to identify the most biologically at-risk cluster (vulnerable compared with nonvulnerable). The effect of the intervention as a function of vulnerability status will be evaluated using repeated measures ANOVA, by comparing vulnerable and nonvulnerable groups. Psychosocial variables will be compared between intervention and nonintervention groups using repeated measures ANOVA, if the conditions for its application are met. A regression analysis will aim to test the mediating role of psychosocial determinants in the improvement of the composite score.

### Sample Size

The number of participants to be included is not based on an a priori calculation. This is due to the absence of data in the literature regarding the effectiveness of a primary prevention program in the military context, measured using a composite criterion that encompasses affect, self-compassion, and hardiness. In order to determine the sample size needed to observe a significant between-group difference, we used the following parameters: a 2-tailed statistical test; a 55% response rate in the intervention group; a 30% response rate in the nonintervention group (significance *P*=.50; power =0.80). If we take into account potential dropouts (estimated at 20%), each group should consist of 58 individuals, making a total of 116 participants.

## Results

The project is currently ongoing, and results are expected to be published by the end of 2024.

## Discussion

This project aims to evaluate an interventional research program for the primary prevention of occupational health problems. The program is as comprehensive as possible and synergistically links physiological, psychological, and social factors. We hope that the integration of biopsychosocial factors, which are suited to the characteristics of the occupational environment, will reinforce the effectiveness of current strategies. The project respects the three pillars of evidence-based prevention: (1) it investigates what is the best evidence and contributes to research-based knowledge; (2) it considers experiential knowledge; and (3) it takes into account the values, preferences, and characteristics of populations and individuals. This type of primary prevention intervention could provide a framework for other interventions that can be modified and adapted to different professional contexts. Data regarding transfer and feasibility collected during our study with operational military personnel could be used in further work to optimize the program for other army corps. Finally, it is imperative to stress that this study adopts an ecological approach, that is, it strives to reflect as closely as possible the real-life experiences of French army soldiers. This approach is essential to obtain information directly applicable to their daily routines and challenges. However, it is essential to recognize that there are potential limitations inherent in this approach, particularly with regard to adherence to the research protocol. Operational constraints within the military environment can sometimes override the ideal execution of the research program. These constraints may require adjustments to the protocol to ensure practicality and feasibility. For example, modifications in the number and frequency of workshops may be necessary to meet service needs and requirements, without compromising operationality. However, the encouraging exploratory results and the interest shown by the military personnel observed by Traber [24] mean that we can be confident that this study will provide a better understanding of the field of PTSD prevention in the military.

The research team is committed to maintaining the integrity of the study while adapting flexibly to these challenges, in order to obtain meaningful and relevant results that will contribute to the well-being and effectiveness of our soldiers in the French army.

## Appendix

Multimedia Appendix 1 Workshop schedule.

Multimedia Appendix 2 Questionnaires used for the primary composite end point.

Multimedia Appendix 3 Genetic polymorphisms involved in stress regulation mechanisms studied in the project.

Multimedia Appendix 4 miRNAs targeting regulatory phenomena established by earlier work and studied in the project.

## Abbreviations

BMS: Burnout Measure Short Version
DRS-15: Dispositional Resilience Scale
HADS: Hospital Anxiety and Depression Scale
ICPH: inner correspondence and peaceful harmony
MSPSS: Multidimensional Scale of Perceived Social Support
OPEX: external operation
PANAS: Positive and Negative Affect Scale
PCL-5: Posttraumatic Checklist-5
PHQ-15: Patient Health Questionnaire
PREPAR: Primary Prevention of Posttraumatic Stress Disorder in Military Professionals
PSS: Perceived Stress Scale
PTSD: posttraumatic stress disorder
TE: traumatic event

## Data Availability

The data sets generated during or analyzed during this study are not publicly available due to French Army data policy but are available from the French Military Health Service at dcssa-paris@sante.defense.gouv.fr upon request.
