# Supplementary material for: Implementation of a Primary Prevention Program for Posttraumatic Stress Disorder in a Cohort of Professional Soldiers (PREPAR): Protocol for a Randomized Controlled Trial
Source: JMIR Res Protoc. 2024 Jan 26;13:e47175. doi: 10.2196/47175 (PMC10858414; doi:10.2196/47175)
Supplement: Multimedia Appendix 1 [file resprot_v13i1e47175_app1.docx]

**Workshop schedule**

| **Session 1** |
| --- |
| **Presentation of the training program, the speakers and objectives** |
| 1. Presentation of the team of trainers, the training schedule, and hearing participants’ expectations in order to create a climate of trust 2. Presentation of objectives compared to other training programs they may have attended. 3. Work on cooperation and support. Creation of pairs to support the practice (training in calm breathing and positive emotions) 4. Motivational interview and implementation of the intention to practice |
| **Session 2** |
| **Physiological stress and biofeedback** |
| 1. Presentation about stress and its physiological manifestations. Distinction between acute and chronic stress. 2. Develop a scenario of stressful events experienced by each person (make a list of 5 or 6 events with a global stress rating) 3. Presentation of the theory of biofeedback and the URGOfeel tool (the application and the sensor) 4. Practice: Calm breathing and positive emotions, Reference to a low-intensity stressful situation, Calm breathing with a focus on potential coping strategies to deal with this situation, Repeat of the same situation with calm breathing, Discussion about feelings and physiological manifestations, Implementation of the practice for the week |
| **Session 3** |
| **Psychological stress** |
| 1. Presentation of the psychological effects of stress and coping strategies 2. Group work on coping flexibility: individual and collective strategies 3. Exposure to moderate stress with the URGOfeel sensor, identification of coping strategies and control over the situation 4. Implementation of the exercise for the week |
| **Session 4** |
| **Work on social support and coping strategies** |
| 1. Presentation of theory on biofeedback and social cohesion 2. Development of a group stress scenario and work on social regulation 3. Emotional regulation strategies (gratitude and interdependence) 4. Implementation of the practice for the week |
| **Session 5** |
| **Introduction to post-traumatic stress** |
| 1. Update on achievements since the beginning of the training 2. Presentation of current knowledge regarding post-traumatic stress disorder 3. De-stigmatizing the disorder 4. Development of an intensely stressful scenario, and a return to calm through emotional and cognitive regulation 5. Feedback and implementation of the practice for the week |
| **Session 6** |
| **Leadership and stigmatization** |
| 1. A review of the week 2. First-hand account from a veteran 3. A presentation by an officer on the value of seeking care 4. Discussion about seeking care, and destigmatizing the disorder 5. Strengthening community values |
| **Session 7** |
| **Empathy and mutual help** |
| 1. A review of the week 2. Presentation and group work on empathy, compassion fatigue and emotional contagion 3. Strengthening feelings of vertical and horizontal cohesion 4. Development of a scenario of emotional contagion, and ways to encourage the return to calm through emotional and cognitive regulation 5. Strengthening self-compassion |
| **Session 8** |
| **Integration of learning from the entire training program** |
| 1. A review of the week 2. Feedback and integration of all the lessons learned: physiological and psychological stress, coping flexibility, post-traumatic stress, care-seeking mechanisms, empathy 3. Strengthening self-compassion 4. Implementation of the practice for the coming months |
