# Supplementary material for: Implementation of a Primary Prevention Program for Posttraumatic Stress Disorder in a Cohort of Professional Soldiers (PREPAR): Protocol for a Randomized Controlled Trial
Source: JMIR Res Protoc. 2024 Jan 26;13:e47175. doi: 10.2196/47175 (PMC10858414; doi:10.2196/47175)
Supplement: Multimedia Appendix 2 [file resprot_v13i1e47175_app2.docx]

**Multimedia Appendix 2. Questionnaires used for the primary composite end point**

| **Name of the questionnaire** | **Measures** | **Number of items** | **How to respond** | **References** |
| --- | --- | --- | --- | --- |
| **The Positive and Negative Affect Scale** | Affect, psychological distress | 20 | Response modality: Very slightly or Not at all (1), Sometimes or a little (2), Occasionally or moderately (3), Quite a bit (4), Extremely (5).  Positive affect scale 1, 3, 5, 9, 10, 12, 14, 16, 17, 19  Negative affect scale 2, 4, 6, 7, 8, 11, 13, 15, 18, 20 | [42] |
| **The Self-Compassion Scale** | Self-compassion | 15 | Response modality: Not at all true (0), Somewhat true (1), Quite true (2), Very true (3)  6 reverse items 3, 4, 8, 10, 11, 14  3 sub-factors:  - commitment: 2, 6, 8, 12, 15,  - control: 1, 4, 7, 10, 13,  - challenge: 3, 5, 9, 11, 14 | [44] |
| **The Dispositional Resilience Scale, DRS-15** | Hardiness | 26 | Response modality: Almost never (1) to Almost always (5).  6 subscales  - Self-help: 5, 12, 19, 23, 26  - Self-judgment: 1, 8, 11, 16, 21  - Human community: 3, 7, 10, 15  - Isola: 4, 13, 18, 25  - Mindfulness: 9, 14, 17, 22  - Over-identification 2, 6, 20, 24 | [43] |
