# Supplementary material for: Implementation of a Primary Prevention Program for Posttraumatic Stress Disorder in a Cohort of Professional Soldiers (PREPAR): Protocol for a Randomized Controlled Trial
Source: JMIR Res Protoc. 2024 Jan 26;13:e47175. doi: 10.2196/47175 (PMC10858414; doi:10.2196/47175)
Supplement: Multimedia Appendix 3 [file resprot_v13i1e47175_app3.docx]

**Genetic polymorphisms involved in stress regulation mechanisms studied in the project.**

- BDNF
- COMT catechol-U-methyltransferase
- Neuropeptide Y: NPY
- Glucocorticoid receptor (NR3C1)
- Mineralocorticoid receptor (NR3C2)
- FKBP5: FK506 binding protein 5
- FKBP4: FK506 binding protein 4
- Serotonin transporter (SLC6A4)
- NPS: neuropeptide S
- NPS receptor (NPSR1)
- Corticotropin receptor (CRHR1)
- Dopamine receptor (DRD2)
- Oxytocin prepropeptide (OXT)
- KL: transmembrane protein involved in aging (KLOTHO)
