# Supplementary material for: Implementation of a Primary Prevention Program for Posttraumatic Stress Disorder in a Cohort of Professional Soldiers (PREPAR): Protocol for a Randomized Controlled Trial
Source: JMIR Res Protoc. 2024 Jan 26;13:e47175. doi: 10.2196/47175 (PMC10858414; doi:10.2196/47175)
Supplement: Multimedia Appendix 4 [file resprot_v13i1e47175_app4.docx]

**miRNAs targeting regulatory phenomena established by earlier work, and studied in the project.**

- miR-132,
- miR-224
- miR-449
- miR-34
- miR-767
- miR-452
- miR-105-1
- miR-105-2
- miR-124a
- miR-18a
- miR15a
- mir-511
- miR-182

All of the above-mentioned targets (polymorphism and miRNA) are likely to change as new knowledge emerges in the field of stress and its consequences. None are studied for diagnostic purposes.
